# Comparing Analgesic Efficacy of a Novel Dual Subsartorial Block Using Two Different Volumes in Patients Undergoing Total Knee Arthroplasty: A Prospective, Double-Blind, Monocentric, Randomised Trial

**DOI:** 10.7759/cureus.20488

**Published:** 2021-12-17

**Authors:** Kartik Sonawane, Hrudini Dixit, Tuhin Mistry, J. Balavenkatasubramanian

**Affiliations:** 1 Anesthesiology, Ganga Medical Centre and Hospitals, Pvt. Ltd, Coimbatore, IND

**Keywords:** procedure-specific ra, opioid-sparing analgesia, total knee replacement, total knee arthroplasty, motor-sparing regional analgesia, dual subsartorial block

## Abstract

Introduction: Total knee arthroplasty (TKA) is a life-changing joint surgery that improves health-related quality of life and functional status. Patients in need of this surgery mostly belong to the geriatric age group with limited functional reserves and multiple co-morbidities requiring utmost perioperative care with the most suitable analgesic modalities. Regional analgesia (RA) should provide effective analgesia while allowing early mobility, reduced opioid consumption, and early discharge. Dual subsartorial block (DSB) is a novel procedure-specific, motor-sparing, and opioid-sparing RA technique for TKA surgeries. Our study compared the analgesic efficacy of the two different combinations of volumes used in DSB.

Methods: This prospective randomized comparative study included patients between 25-75 years of age of American Society of Anesthesiology (ASA) I-II grades who underwent an elective cemented unilateral total knee replacement performed via medial approaches under neuraxial anesthesia. A total of 104 patients were divided into two equal groups based on the local anesthetic (LA) volumes (Group A 10/20 ml and Group B 20/10 ml) used in the DSB. Postoperative pain scores (using a visual analog scale) and quadriceps strengths (using neurological exam), and opioid consumption were measured at regular intervals till discharge.

Results: Most patients (71.2%) remained pain-free and comfortable until discharge, while 28.8% complained of pain within 12 hours of DSB. Mean quadriceps strength remained almost normal (4-5/5) until the discharge with no incidences of buckling or fall in either group. Over time, the postoperative trend between the groups showed a significant difference for dynamic pain (p = 0.002) and quadriceps strength (p = <0.001). There was an insignificant difference (p = 0.161) between the groups regarding opioid consumption, with the median oral morphine equivalent of zero in both groups.

Discussion: The effective analgesic coverage of DSB is based on the involvement of all innervations of the procedure-specific pain generators of TKR surgeries. The specific focus on selective sensory innervations and the type/volume of the LA used makes DSB a motor-sparing RA alternative that facilitates early mobility and discharge. It can provide effective postoperative analgesia without compromising the motor strength of the quadriceps muscle when administered in either 10/20 or 20/10 volumes.

## Introduction

Osteoarthritis (OA) is the leading cause of disability in older adults [1]. It is the fifth-highest cause of years lost to disability in high-income countries and the ninth highest cause in low and middle-income countries [2]. Approximately 9.6% of men and 18% of women over the age of 60 years suffer from symptomatic OA worldwide, while 80% of those affected have restricted movements and 25% cannot perform major daily activities of life [3]. Its increasing prevalence is due to the growing geriatric population and associated risk factors such as obesity and sedentary lifestyle. Overall, it leads to physical disabilities through pain and loss of functionality, reducing the quality of life and increasing the risk factors for further morbidity. Radiographically, knee OA is found in around 30% of the elderly population, accounting for 50% of the total disease burden on the musculoskeletal system [4]. Such a population should receive enhanced perioperative care that includes the most appropriate analgesic modalities to improve quality of life.

A well-functioning knee joint makes a significant contribution to the quality of life. Total knee arthroplasty/replacement (TKA/TKR) is the ultimate treatment option for end-stage OA of the knee joint to improve health-related quality of life and functional status [5]. It is the most commonly performed life-changing surgical procedure of the modern world [6,7]. The advent of modern TKR surgeries demands optimal perioperative analgesia that favors early mobility and discharge. Extensive research has been done to map the complex innervation of the knee joint and find the appropriate regional analgesia (RA) techniques suitable for TKR [8-25]. The ultimate goal of RA in TKA as an adjunct to multimodal analgesia (MMA) is to provide effective analgesia while allowing early mobility (motor-sparing), reduced opioid consumption (opioid-sparing), and early discharge suitable for enhanced recovery after surgery (ERAS) protocol.

The dual subsartorial block (DSB) is a novel procedure-specific and motor-sparing RA technique that may reduce overall postoperative opioid consumption when used in conjunction with MMA [26-28]. In our hospital, the DSB is being used in patients scheduled for planned elective TKA as a cornerstone of postoperative analgesia protocols in combination with systemic analgesia since 2017. Prior to 2017, the standard of care was a femoral nerve block (FNB) in conjunction with systemic non-opioid and opioid analgesics. The effective postoperative analgesia, reduced rescue analgesic demands, and the ERAS suitability made DSB the block of choice for all TKR patients in our institution.

We aim to compare the analgesic effects and functional consequences of two different volumes used in the DSB in consecutive patients who met the inclusion criteria. We hypothesize that DSB would provide the desired procedure-specific, motor-sparing, and opioid-sparing analgesia when utilized in 10-20 ml volumes for each injection. We propose a new paradigm and an alternative RA technique to improve the quality of post-TKR analgesia until the discharge, facilitating ERAS.

## Materials and methods

Patients

The inclusion criteria of our study comprised patients of either sex between 25-75 years of age with American Society of Anesthesiologists (ASA) I-II grades who underwent an elective cemented unilateral TKA/TKR with varus knee or mild valgus deformity performed via medial approaches by a single arthroplasty surgeon under neuraxial anesthesia.

The exclusion criteria of our study included patients with ASA III/IV grade, severe valgus deformity, acute/chronic kidney disease, neurological deficit, cognitive dysfunction, any contraindications for the neuraxial blockade, coagulopathy, chronic opioid consumption (daily or almost daily use of opioids for > three months), operative limb neuropathy, hypersensitivity and/or allergies to local anesthetic (LA) or any of the study medications, or patients who underwent TKA other than medial approaches, bilateral TKA, or patient refusal in study participation.

Sample size estimation

The number of participants to be included was calculated based on the pilot study [26] conducted with reported mean pain scores (SD) of 0.8 (1.1) in the 10/20 group and 1.6 (1.14) in the 20/10 group.

The sample size required in each arm of the study was calculated according to the formula:

Sample size N = 2(zα + z1-β )­2 σ2 / δ2

Where σ (Pooled SD) = 1.12, δ (Difference of Means) = 0.8, Type I error (α) = 5%, Zα = 1.96, Type II error (β) = 5%, Power (1 - β) = 95%, and Z1-β = 1.65. So, based on the formula given above, using the mentioned values, the sample size required is: 2*(1.96 + 1.65)2*1.122/0.82 = 51.08. Thus, assuming 95% power and 95% confidence interval, the inclusion of 52 patients in each group (total number of 104 patients) was considered sufficient to observe a significant difference between both groups.

Details of study design

After approval by the Institutional Review Board (IRB/GH/Anaes/02/2019/003) and written informed consent, 104 patients were allocated into two equal groups (52 patients each) based on the volumes used in the DSB.

1. Group A: 10 ml in the distal femoral triangle and 20 ml in the adductor canal.

2. Group B: 20 ml in the distal femoral triangle and 10 ml in the adductor canal.

This clinical trial was registered in the Clinical Trial Registry of India (http://ctri.nic.in/Clinicaltrials/pdf_generate.php?trialid=26029&EncHid=72403.49104&modid=1&compid=19%27,%2726029det%27) as a prospective trial (No. CTRI/2019/04/018733, dated 24/04/2019). It was conducted between May 2019 (first case enrolment from 01/05/2019) and May 2021 (about 24 months) as a prospective, double-blind, monocentric study and randomized comparative trial.

Methods of randomization and blinding

Computer-generated randomization. Aside from the researchers performing the blocks, all other investigators, anesthetists, surgeons, physical therapists, nurses, and the study participants were blinded to the randomization of each subject.

Patient preparation

Preoperatively, approximately 10 ml/kg Ringer’s lactate solution was given to the patients according to their hemodynamics after securing intravenous access. All patients were premedicated with intravenous 0.3 mg ramosetron and 8 mg dexamethasone as an antiemetic medication.

Intraoperatively, routine anesthetic monitoring was conducted using non-invasive blood pressure, pulse oximetry, and electrocardiography. All patients received spinal anesthesia performed in standard fashion (L3-L4) using 10-15 mg of hyperbaric bupivacaine 0.5%. All patients were sedated by 0.01 mg/kg midazolam, when necessary, intraoperatively. Intraoperative tourniquet pressure and duration were noted.

Immediately after completion of surgery and application of dressing, an experienced anesthesiologist (with more than five years of experience in ultrasound-guided RA techniques) performed ultrasound-guided DSB using prepared LA solution (0.2% ropivacaine + 8 mg dexamethasone).

Postoperatively, all patients were kept under observation in the high dependency unit for 24 hours for postoperative monitoring and pain management. During the entire hospital stay, the patients were continued on the pharmacological regimen (Table 1).

**Table 1:** Perioperative pharmacological regimen

Timing	Medications
On the night before surgery:	Oral paracetamol 1 gm + pantoprazole 40 mg + pregabalin 75 mg + aceclofenac 100 mg.
One hour before surgery:	Intravenous ramosetron 0.3 mg + tranexamic acid 1 gm + paracetamol 1 gm.
Intraoperatively/Before skin incision:	Intravenous ketorolac 30 mg + dexamethasone 8 mg with adequate maintenance fluids.
Postoperatively in the first 24 hrs:	Intravenous paracetamol 1 gm 6 hourly, ketorolac 30 mg 12 hourly, and pantoprazole 40 mg od. Buprenorphine transdermal patch 5-10 mg for 7 days. Night sedation with intramuscular Butadol 1mg and Phenergan 12.5 mg.
Postoperatively after 24 hrs:	Oral paracetamol 1 gm qid + pantoprazole 40 mg od + pregabalin 75 mg hs + aceclofenac 100 mg bd + Ecosprin 150 mg od.

DSB technique

For DSB, the LA solution was deposited under the sartorius muscle (subsartorial regions) with two injections (dual) that combined two subsartorial blocks: the distal femoral triangle block (FTB) and the adductor canal block (ACB). The DSB was given in three steps [27,28].

Identification of the Apex of FT

**Figure 1:**
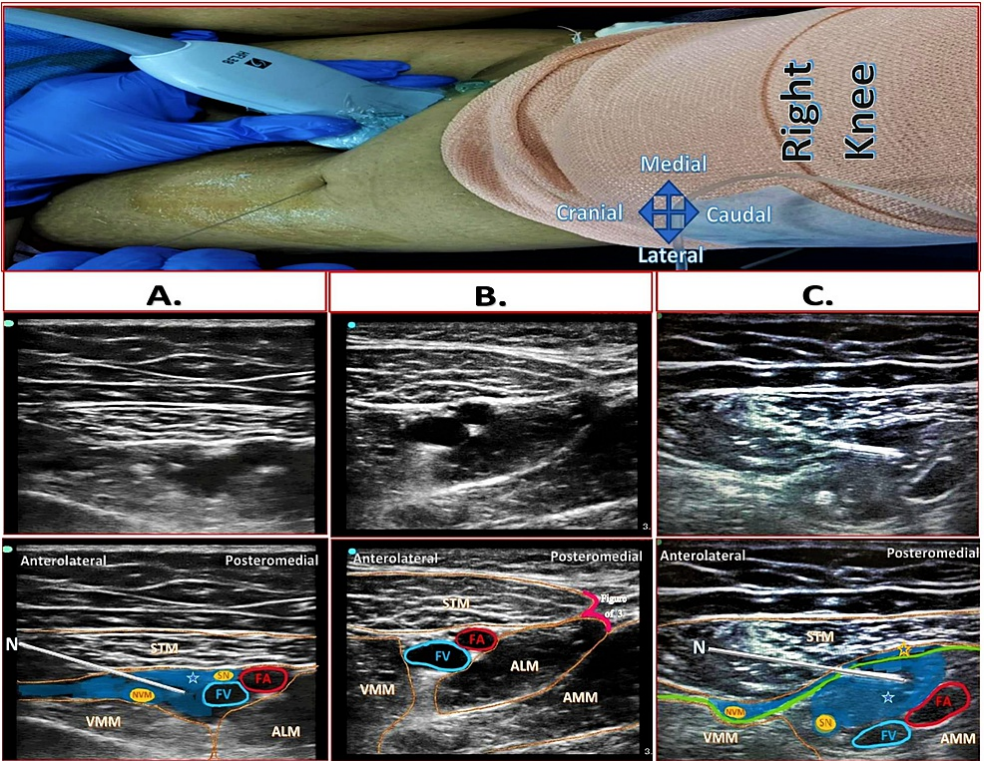
Probe position and sonoanatomy of Dual Subsartorial Block A: Distal femoral triangle block B: Apex of the femoral triangle – Intersection point of medial borders of ALM and STM forming “figure of 3” also called as “kissing sign” C: Adductor canal block (STM: Sartorius muscle, ALM: Adductor longus muscle, VMM: Vastus medialis muscle, AMM: Adductor magnus muscle, FA: Femoral artery, FV: Femoral vein, SN: Saphenous nerve, NVM: Nerve to vastus medialis, Blue area: Local anesthetic drug spread, Green line: Vasoadductor membrane, Blue star with blue border: Drug spread below VAM in adductor canal, Blue star with orange border: Drug spread above VAM) Source: Sonawane K, Dixit H, Mistry T, Balavenkatasubramanian J (2021) Anatomical and Technical Considerations of “Dual Subsartorial Block” (DSB), A Novel Motor-sparing Regional Analgesia Technique for Total Knee Arthroplasty. Open J Orthop Rheumatol 6(1): 046-056. https://dx.doi.org/10.17352/ojor.000038

A high-frequency linear ultrasound probe (10-12 Hz; SonoSite Edge; SonoSite Inc., Bothell, WA, USA) was placed anteromedially over the mid-thigh location (Figure 1). The apex of the femoral triangle (FT) was identified as an intersecting point of medial borders of sartorius and adductor longus muscles, forming a sign of “3” or “Kissing Sign” (Figure 1B).

First Injection (Distal FTB)

The probe was moved 1-2 cm proximal to the apex of FT and kept over the distalmost part of the FT. The sonoanatomy revealed sartorius muscle (STM) above the femoral vessels, the vastus medialis muscle (VMM) anterolaterally, and adductor longus muscle (ALM) posteromedially. Between STM and VMM, hyperechoic saphenous nerve (SN) lateral to the femoral artery (FA) and the nerve to vastus medialis (NVM) lateral to the SN were identified. A 100-mm, 22-gauge Stimuplex A needle (B. Braun Medical Inc., Melsungen, Germany) was inserted in-plane from the lateral-to-medial direction in the plane between STM and VMM. The LA mixture was deposited targeting SN and NVM, separating the plane between VMM and STM (Figure 1A).

Second Injection (ACB)

After the first injection, the probe was moved distally to re-identify the apex of the FT and then placed 1-2 cm distal from it over the proximal AC. The bilayered appearance of the lower border of STM was noted due to the vasoadductor membrane (VAM). The LA mixture was deposited besides FA under the VAM, sometimes resulting in compression of the FA (Figure 1C).

Patient assessment and data collection

**Figure 2:**
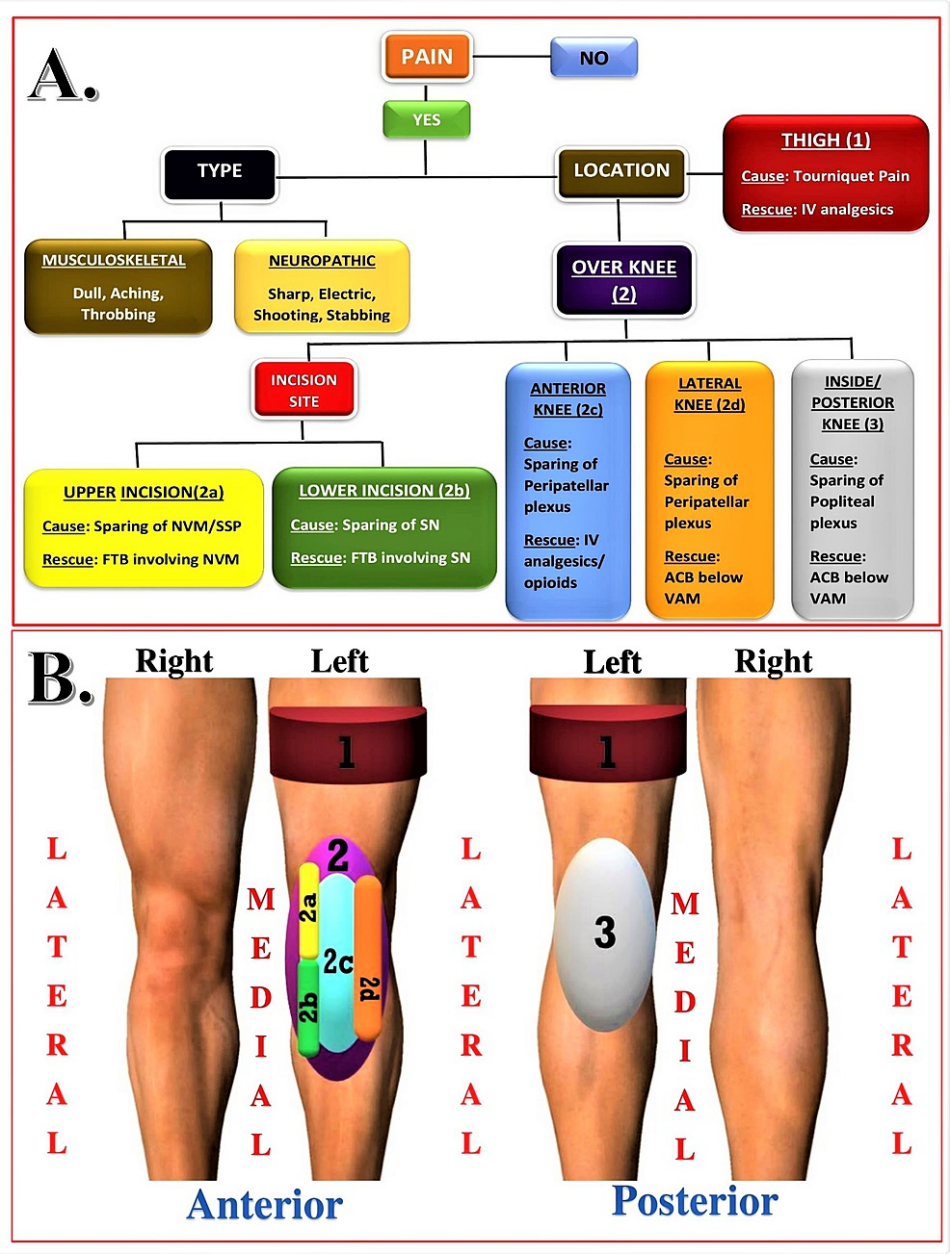
Postoperative pain assessment and rescue analgesia plan A: Rescue analgesia as per the location of the pain B: Pain location mapping of postoperative knee pain (IV: intravenous,  NVM: nerve to vastus medialis, SSP: subsartorial plexus, FTB: femoral triangle block, SN: saphenous nerve, ACB: adductor canal block, VAM: vasoadductor membrane)

Postoperative pain scores (resting and dynamic) were measured using a visual analog scale (VAS) at regular intervals till the discharge of the patient. The quadriceps strengths were also measured periodically by a neurological exam, based on a 6-point (0-5) scale from the Medical Research Council (MRC) (0 = no contraction, 1 = flickering or signs of contraction, 2 = active movement without gravity, 3 = active movement against gravity, 4 = active movement against gravity and resistance, 5 = normal power) [29]. The consumption of additional opioids (fentanyl) as rescue analgesia was recorded and converted to oral morphine equivalent in milligrams. The incidence of side effects/complications in the postoperative period, the patient satisfaction scores, and the duration of post-surgery hospital stay were recorded. The sensory duration of the block was defined as the time taken from completion of the block till the first call for analgesia or VAS ≥ 5. The rescue analgesia was determined as per the pain locations postoperatively (Figure 2). Buckling was defined as a sudden and unintentional loss of postural strength and balance seen by the staff, requiring the patient to use self-support to prevent a fall [30].

Primary and secondary outcomes were as shown in Table 2.

**Table 2:** Primary and secondary outcomes of the comparative trial DSB: Dual Subsartorial Block

Primary and secondary outcomes
Primary outcomes:
Observing the analgesic efficacy of DSB between the groups using the postoperative visual analog score (VAS) at specific intervals till the discharge of the patient.
Measuring the operated limb quadriceps strength between groups using 6 points (0-5) scale every six hourly postoperatively till discharge.
Secondary outcomes:
Calculating additional postoperative opioid (fentanyl) consumption between groups till discharge.
Determining the sensory duration of the block.
Determining the most common location of nerve to vastus medialis and saphenous nerve during ultrasound scanning.
Determining the depth of the target structures.
Determining the total postoperative length of hospitalization.
Determining the effect of tourniquet pressure and duration on post-block analgesia.
Localizing postoperative pain if VAS >5 using 1-3 codes [1 = thigh, 2= anterior knee (2A = upper part of incision, 2B= lower part of incision, 2C= anterior knee, 2D= lateral knee), 3 = posterior knee, and mixed locations].
Identifying the likely causes of pain (tourniquet, physiotherapy, inadequate block, and unknown).
Recording incidences of complications, if any.
Determining patient’s overall experience score during discharge using 0-5 score (0 = worst, 1= very bad, 2 = bad, 3 = good, 4 = very good, 5 = best).

Statistical methods

**Figure 3:**
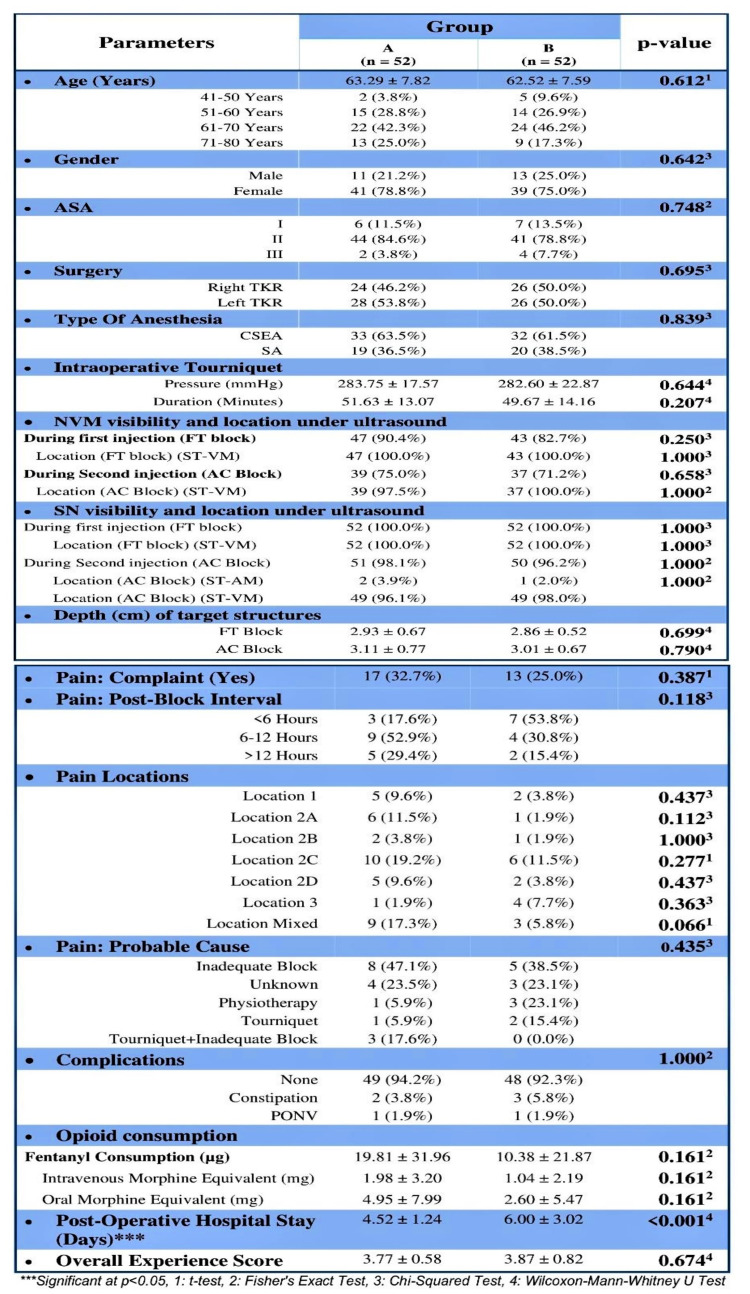
Patient’s demographic details and characteristic chart of comparative parameters between the groups

**Figure 4:**
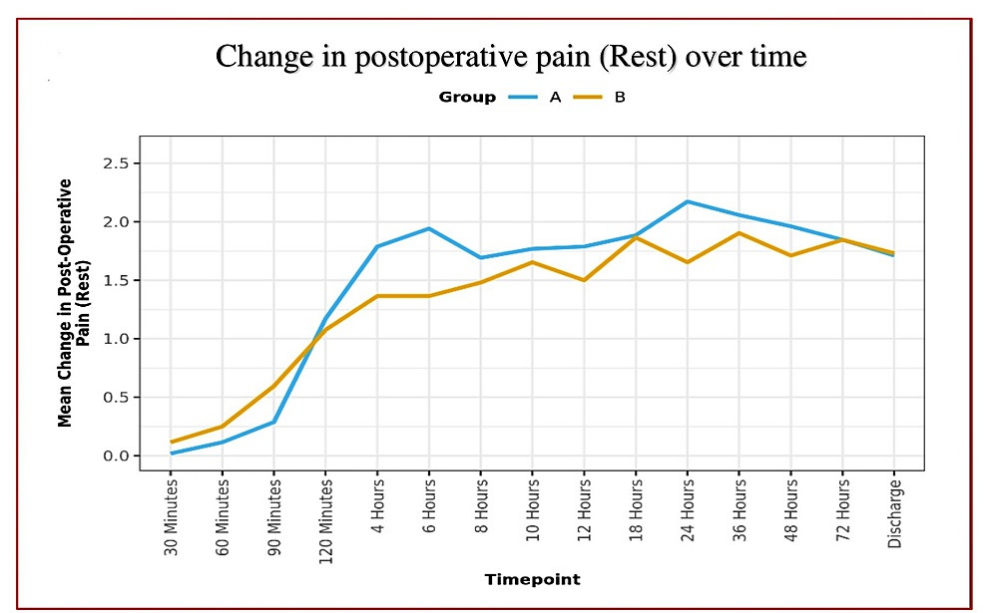
Line diagram depicting postoperative rest pain scores over time

**Figure 5:**
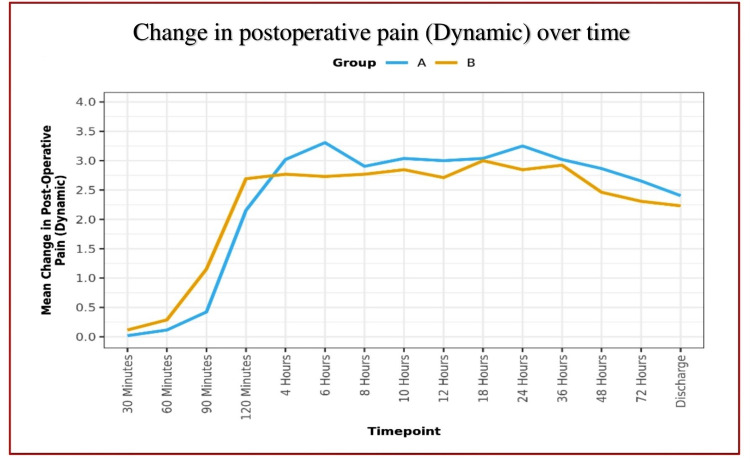
Line diagram depicting postoperative dynamic pain scores over time

**Figure 6:**
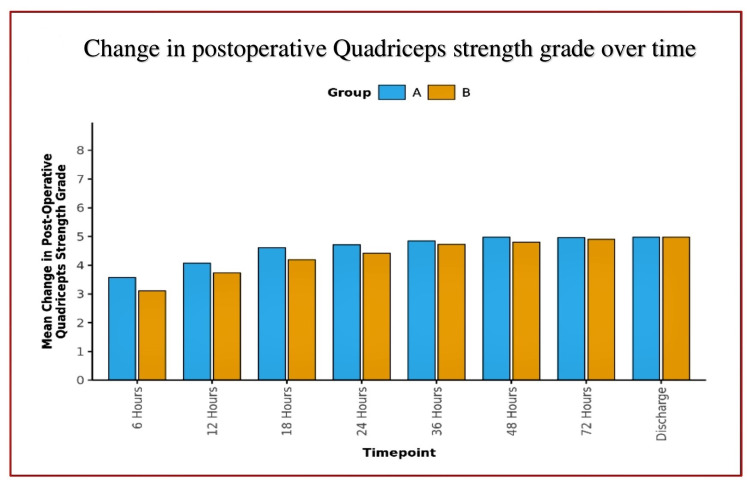
Bar diagram depicting postoperative quadriceps strength grades over time

**Figure 7:**
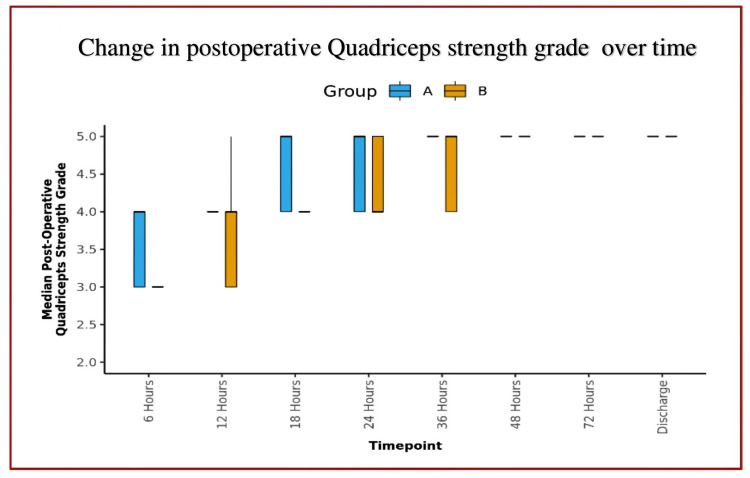
The Box-and-Whisker plot showing the distribution of postoperative quadriceps strength grade over different time points (In each box, the middle horizontal line represents the median postoperative quadriceps strength grade, the upper and lower bounds of the box represent the 75th and the 25th centile, respectively, and the upper and lower extent of the whiskers represent the Tukey limits for postoperative quadriceps strength grade at each of the timepoints respectively)

**Figure 8:**
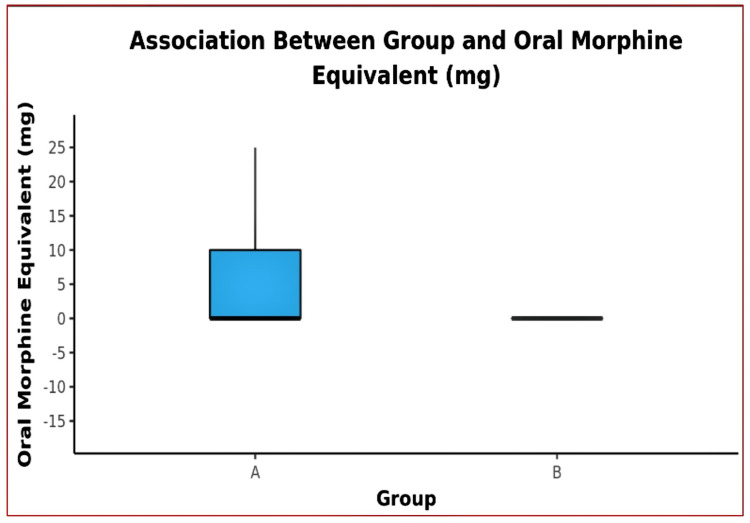
The Box-and-Whisker plot showing consumption of oral morphine equivalents (mg) between groups (The middle horizontal line represents the median oral morphine equivalent (mg), the upper and lower bounds of the box represent the 75th and the 25th centile respectively, and the upper and lower extent of the whiskers represent the Tukey limits for oral morphine equivalent (mg) in each of the groups)

**Figure 9:**
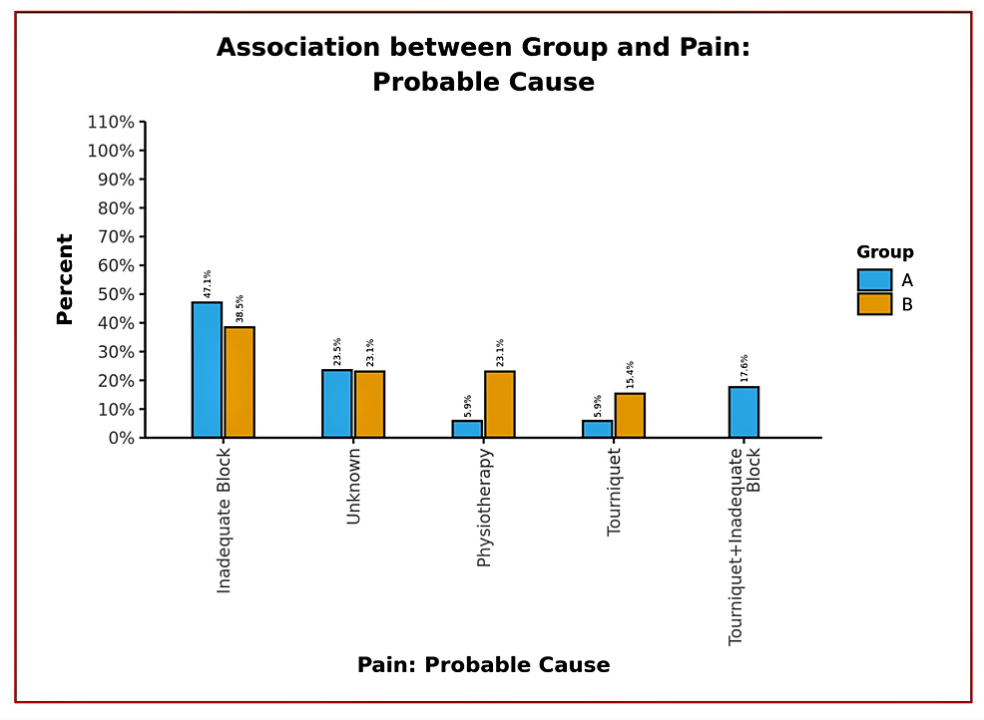
Bar diagram showing probable cause of pain between groups

**Figure 10:**
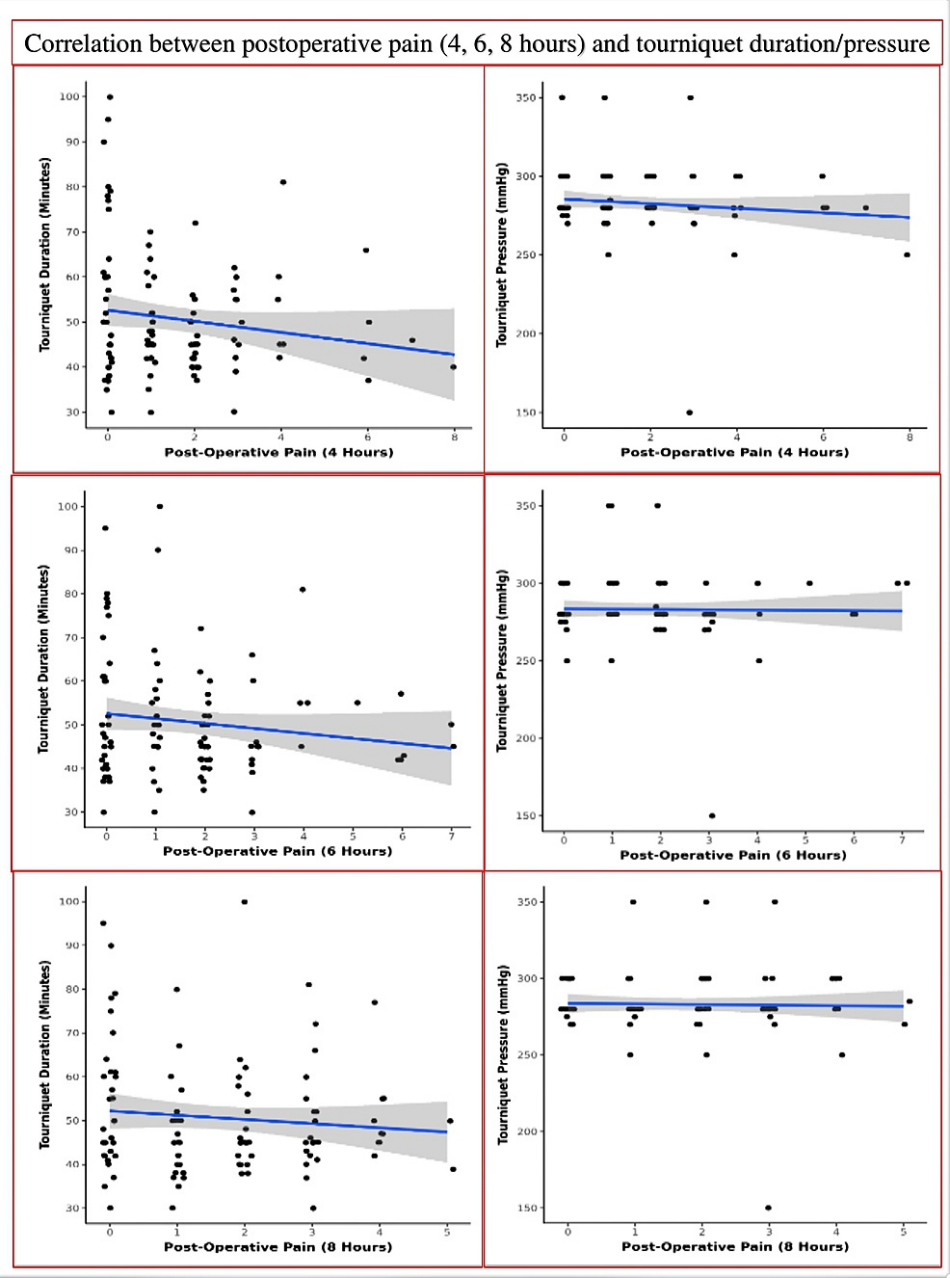
Scatter plot diagram showing a correlation between postoperative pain (at 4, 6, and 8 hours) and tourniquet pressure (mm of Hg) and duration (minutes) (Individual points represent individual cases. The blue trendline represents the general trend of correlation between the two variables. The shaded grey area represents the 95% confidence interval of this trendline)

The data was encoded and recorded in an MS Excel spreadsheet program. The statistical analysis was carried out with SPSS v23 (IBM, Armonk, NY, USA). Descriptive statistics were generated in the form of means/standard deviations and medians/interquartile ranges (IQRs) for continuous variables and frequencies and percentages for categorical variables. The data were presented graphically using histograms/box and whisker plots/column charts for continuous data and bar charts/pie charts for categorical data (Figures 3-10). Group comparisons for continuously distributed data were performed using the independent sample t-test (two groups) and one-way ANOVA (greater than two groups). For non-normally distributed data, suitable non-parametric tests such as the Wilcoxon test/Kruskal-Wallis test were used. Categorical variables were assessed using the chi-square test or Fisher exact test. The value of p <0.05 was considered to be statistically significant.

## Results

**Figure 11:**
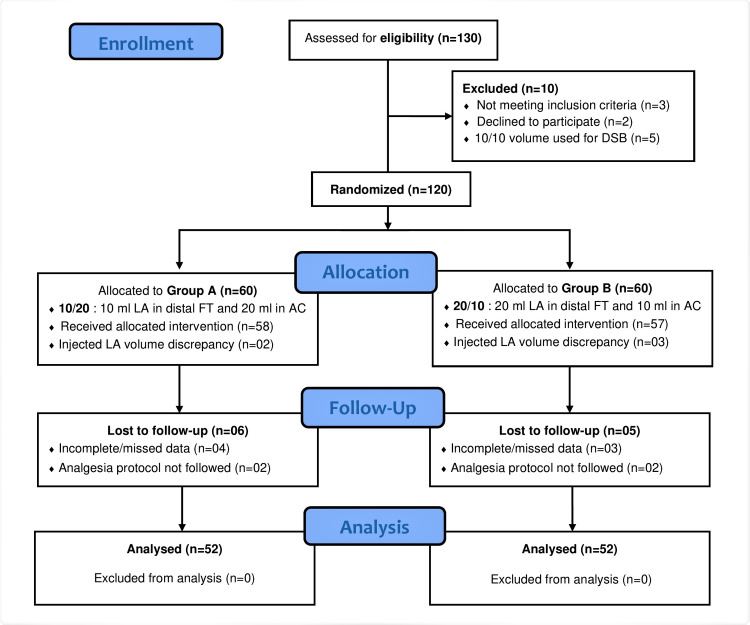
Consort diagram showing assignment of the patients in study groups (DSB: dual subsartorial block, FT: femoral triangle, AC: adductor canal, LA: local anesthetic)

The assignment of patients in study groups is shown in Figure 11. A total of 104 consecutive patients were included: 52 in Group A and 52 in Group B. This population was predominantly female (76.9%, 80 patients), with a median (IQR) age of 63 (58-70) years. Preoperative characteristics (age, gender, ASA status, type of anesthesia, co-morbidities) were comparable in both groups. The other measured parameters and complications showed no significant differences between the groups. The main results of the study are presented in Table 3.

**Table 3:** Main outcomes of the comparative study (DSB: Dual Subsartorial Block, IQR: interquartile range)

Main outcomes and observations	
Analgesic efficacy of DSB between groups:	In Group A, the mean postoperative rest pain increased from 0.02 (at the 30 minutes) to 2.17 (at the 24 hours) and then decreased to 1.72 at the discharge; whereas dynamic pain increased to 3.31 (at the 6 hours), and then it decreased to 2.40 at the discharge. This change was statistically significant [Friedman Test: χ2 = 245.2 (rest) and 419.2 (dynamic), p = <0.001].
	In Group B, the mean postoperative rest pain increased from 0.12 (at the 30 minutes) to 1.90 (at the 36 hours) and then decreased to 1.73 at discharge, whereas dynamic pain increased to 3.00 (at 18 hours) and then decreased to 2.23 at the discharge. This change was statistically significant [Friedman Test: χ2 = 213.5 (rest) and 353.3 (dynamic), p = <0.001].
	Over time, the postoperative pain trend showed no significant difference between the two groups (p = 0.330) for rest pain but a significant difference for dynamic pain (p = 0.002).
Quadriceps strength between groups:	Both groups differed significantly in postoperative quadriceps strength grade at 6, 12, 18, 24, and 48 hours.
	The mean postoperative quadriceps strength grade increased from a minimum of 3.58 (Group A) and 3.12 (Group B) at 6 hours to a maximum of 4.98 (both groups) at discharge.
	Both changes were statistically significant for both groups [Friedman Test: χ2 = 272.1 (Group A) and 277.3 (Group B), p = <0.001].
	Over time, the postoperative quadriceps strength grade trend showed a significant difference between the groups (p = <0.001).
Additional opioid consumption:	The median (IQR) of oral morphine equivalent consumption was 0 (0-10) mg in Group A and 0 (0-0) mg in Group B.
	No significant difference between the groups in terms of opioid consumptions (W = 1522.500, p = 0.161).
Sensory duration of block:	Of 104 participants, 71.2% participants (n=74) remained pain-free until discharge.
	The median (IQR) interval of post-block pain was 8 (6-14) hours in Group A and 5 (4-10) hours in Group B.
	The sensory duration of the block was <6 hours for 10 participants [3 (17.6%) of Group A and 7 (53.8%) of Group B], 6-12 hours for 13 participants [ 9 (52.9%) of Group A and 4 (30.8%) of Group B], and >12 hours for 7 participants [ 5 (29.4%) of Group A and 2 (15.4%) of Group B].
	No significant difference between the groups regarding post-block pain intervals (W = 152.000, p = 0.083).

We found effective equivocal analgesia provided by DSB in both groups. The majority of the patients remained comfortable and pain-free till discharge, with a mean VAS score of less than 3 in both groups (Figures 4-5). In remaining patients (28.8%) who complained of pain within 12 hours after DSB, various causes of pain were determined as per the location of postoperative pain. We found no significant difference between the various groups in terms of probable causes (χ2 = 4.719, p = 0.435) and distribution of pain at all locations: Location 1 (χ2 = 1.378, p = 0.437), Location 2a (χ2 = 3.829, p = 0.112), Location 2b (χ2 = 0.343, p = 1.000), Location 2c (χ2 = 1.182, p = 0.277), Location 2d (χ2 = 1.378, p = 0.437), Location 3 (χ2 = 1.891, p = 0.363), and Location Mixed (χ2 = 3.391, p = 0.066).

The mean quadriceps strength remained almost normal (4-5/5 grade) until the discharge of the patients (Figures 6-7), with no incidences of buckling or fall in both groups. The median of additional opioid consumption of both groups was zero (Figure 8, Table 3). We also found that the most common location of NVM and SN is between STM-VMM during FT injection. However, the location of SN varied as per the site of injection (proximal, mid, or distal) in the AC. The mean depth of the target structures was <3.5 cm at both injection sites (FT and AC) of DSB. The percentage of an inadequate block as the likely cause of pain in patients who developed significant pain was higher in Group A than in Group B (Figure 9).

The mean (SD) of tourniquet pressure (mmHg) in Group A was 283.75 (17.57), and Group B was 282.60 (22.87). The mean (SD) of tourniquet duration (minutes) in Group A was 51.63 (13.07), and Group B was 49.67 (14.16). There was no significant difference between the groups regarding tourniquet pressure (W = 1290.000, p = 0.644) or tourniquet duration (W=1546.000, p=0.207).

We found a weak negative but insignificant correlation between postoperative pain and tourniquet duration at four hours (rho = -0.1, p = 0.294), six hours (rho= -0.1, p= 0.319), and eight hours (rho = -0.06, p = 0.559). We also found weak positive but insignificant correlation between postoperative pain and tourniquet pressure at four hours (rho = -0.04, p = 0.711), six hours (rho = 0.02, p = 0.854), and eight hours (rho = 0.04, p = 0.668) (Figure 10).

## Discussion

In our study, we compared two different volumes of LA in two different components of DSB (distal FT and AC) to assess its analgesic efficacy when used as an adjunct to MMA in the postoperative period. The DSB can also be classified as a precision RA technique as it targets specific neural elements using LA of fixed volumes and concentration at fixed locations. Optimal analgesia provided by DSB as an important MMA component helped reduce the additional opioid consumption making DSB an opioid-sparing RA option. We found significantly retained quadriceps muscle strength in both groups with no evidence of buckling or falls. It allowed our patients to continue physiotherapy and pain-free mobilization, promoting early hospital discharge. Thus, making the DSB a motor-sparing RA alternative among available techniques.

DSB is also classified as a procedure-specific block as it covers both anterior and posterior knee innervations of all pain generators involved in the surgical dissection [26-28]. The pain-generating components before and after surgery are different. Before surgery, intraarticular components outweigh the pain generation due to inflammation from the osteoarthritic changes in the joint [26-28,31]. After surgery, all intraarticular pain-generating components are removed and replaced by nonneural and nonvascular implants. The postoperative pain-generating components mainly include skin/subcutaneous tissues over the incision area, medial retinaculum, periosteal rim of the cut bones, remnant of the anterior joint capsule, severed nerves, microfractures, and postsurgical inflammation. Therefore, anterior components predominate the intra-articular components in postoperative pain generation due to the surgical dissection in the previously healthy tissues. The procedure-specific analgesic coverage of the DSB is primarily intended for TKR surgeries with the medial approaches. For other approaches, the analgesic coverage of DSB may be limited due to completely different procedural innervations requiring different neural blocks.

Pain scores (rest and the dynamic) of both groups showed effective and equivocal analgesia throughout the postoperative period, possibly due to the broad coverage of all procedure-specific innervations by DSB. The first injection of DSB targets SN and NVM directly and the subsartorial plexus (SSP) and peripatellar plexus (medial half) indirectly through the drug distribution. Thus, it covers the innervation of the anterior knee components [27,28]. The second injection of DSB targets popliteal plexus indirectly due to distal drug spread. Thus, it covers the innervation of the intraarticular and posterior knee components [27,28]. The patterns of the drug spread and the analgesic coverage of DSB are consistent with the finding of dye studies at the level of FT and AC [32-39].

**Figure 12:**
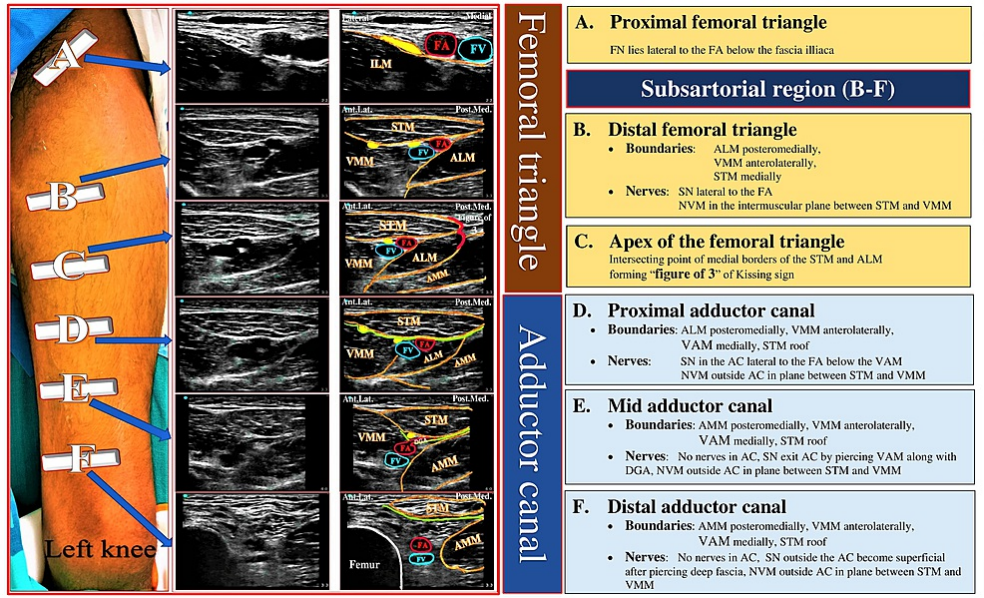
Sonoanatomy at various levels of the thigh and demarcation of the femoral triangle and adductor canal (FA: femoral artery, FV: femoral vein, ILM: iliacus muscle, Ant. Lat.: anterolateral, Post. Med.: posteromedial, STM: sartorius muscle, VMM: vastus medialis muscle, ALM: adductor longus muscle, AMM: adductor magnus muscle, DGA: descending genicular artery, SN: saphenous nerve, NVM: nerve to vastus medialis, VAM: vasoadductor membrane, AC: adductor canal, Yellow dots: nerves)

The desired results of the DSB depend on the understanding of the anatomy of two prominent landmarks in the thigh (FT and AC) separated by the FT apex to locate target-specific innervations [26-28]. The STM can be seen as a common muscular landmark under ultrasound from the distal FT to the distal AC regions. Therefore, the area below the STM in these regions can generally be referred to as the subsartorial area [26-28]. The outcomes of the subsartorial blocks may vary depending on the point of administration due to different anatomical relationships of the neural elements (Figure 12). We found a consistent location of the NVM between the STM and VMM in both FT and AC regions. We chose to block the NVM in the FT region as it lies in a separate fascial sleeve above the VAM in the AC region, which may resist the blockade [36,37,40]. The distal drug spread of FT injection into the AC region above the VAM was also clearly seen while giving AC injection. Proximal tracking of the drug spread after FT injection showed no involvement of the FN under ultrasound, which is consistent with the observations of our ongoing cadaveric study on DSB. For all the participants, we could use a high-frequency ultrasound probe to obtain a high-resolution image to deposit LA solution in the correct location due to the shallow depth (<3.5 cm) of the target nerves (SN and NVM) at both locations (FT and AC).

Another important outcome of our study was to assess the motor-sparing effect of DSB at regular intervals postoperatively until discharge of the patient. We found significantly preserved quadriceps muscle strength in both groups in all study time points without any incidences of fall or buckling after DSB administration. However, there was an initial decrease in quadriceps strength in both groups during the first recording at the sixth hour after DSB, possibly due to the residual effects of the neuraxial anesthesia. We believe that the limited range of motion due to chronic osteoarthritic changes in the knee joint can lead to quadriceps weakness or reduced baseline quadriceps strength. However, we could not record preoperative (baseline) quadriceps strength to compare the changes before and after surgery, as such comparison would be beyond the scope of this study. Quadriceps strength may also decrease following knee surgery or commonly used RA techniques. Some studies documented less decreased quadriceps power following ACB than FNB [41], while others documented higher quadriceps power following midthigh ACB than FNB [42,43]. However, many studies have confirmed that the mid-thigh location is likely the FT region rather than the AC [44,45].

The motor-sparing effect of DSB is mainly due to its components (distal FTB and ACB), selective motor sparing neuronal targets, and choice of LA used. Distal FTB may decrease quadriceps strength due to proximal drug spread involving the femoral nerve (FN) [17,40,46,47]. A combination of distal FTB and infiltration between the popliteal artery and capsule of the knee joint (iPACK) and our study showed no mobilization failure due to motor block [48,49]. The other component, the ACB, is known for its quadriceps preservation and enhanced early ambulation compared to FNB and significantly lower incidence of buckling (three vs. 17 patients, p = 0.004) [30]. Grevstad et al. noted an increase in the quadriceps function following ACB in patients with severe post-TKR pain, representing the combined analgesic and quadriceps-sparing property of ACB [50].

In DSB, we selectively targeted terminal nerves preserving motor branches that further contributed to the motor-sparing effect of this block. Johnston et al. [51], in their study about ACB, determined a minimum effective volume of 0.5% ropivacaine in 50 % of the subjects needed for 30% decrease in quadriceps power (ED50) as 46.5ml whereas, in 95% of the subjects (ED95), it was 50.32 ml. The volumes used (10/20 and 20/10 ml) in our study in both groups showed no clinical evidence of quadriceps weakness, as they were within limits determined by Johnston et al. We use the LA agent type and concentration (0.2 % ropivacaine) known for its motor-sparing effect [52,53] along with an adjuvant (dexamethasone) that causes prolonged analgesia (mainly sensory) duration [54,55]. Therefore, owing to anatomical determinants (targeted sensory innervations) and pharmacological factors (LA type/volumes), the DSB can be deemed a motor-sparing RA technique.

The other motor-sparing RA options for the TKR included FTB, ACB, iPACK, modified 4-in-1 block, and local infiltration analgesia (LIA). The FTB or ACB alone may not cover the innervations of all procedure-specific pain-generating components, resulting in ineffective or partially effective analgesia. In ultrasound-guided modified 4-in-1 block [56], 5-7 ml of drug is injected at the level of the adductor hiatus to block NVM after PNS identification, and 20-25 ml of drug is injected under the VAM around the femoral vessels. The FT apex is a relatively easier identification landmark (for DSB) on ultrasound than the descending genicular artery (for modified 4-in-1 block), thus making the DSB an easier block to administer, learn and teach. Also, we chose to target the NVM in the distal FT region, where it is bare and not covered by any additional fascial sheath. Although the overall effect of the second injection of DSB is similar in all AC regions (proximal, mid, or distal), we recommend proximal AC injection to avoid proximity to the surgical field, which may be worrisome for the surgeons. The modified 4-in-1 block is usually given preoperatively, potentially delaying the surgery and truncating the analgesia duration. It may also spare the SSP overlying the VAM in the AC. However, due to distal drug spread in the AC region below sartorius and above VAM, the SSP may consistently be involved in the distal FT injection of DSB (Figure 1C). Combining ACB with iPACK provided equivalent analgesia to FNB with or without iPACK and improved physical therapy performance, allowing earlier hospital discharge [57]. We believe that giving ACB with iPACK will have no added advantage as they block the same plexus (popliteal plexus) [58] due to the drug distribution in the popliteal region. LIA is a promising option for postoperative pain relief, reducing total narcotic consumption in TKR surgery [59,60]. However, the use of steroids and ketorolac in LIA potentially increase the risk of intra-articular infection [61] and the possibility of renal and gut toxicity, respectively [59]. Intra-articular LIA catheters are associated with an increased risk of infection, chondrocyte damage, and drug intoxication [62,63]. The chondrotoxic effect of LIA and the possibility of infections may lead to joint loosening requiring redo surgery in long-term follow-ups. Other drawbacks of LIA are surgeon-dependent analgesia coverage and limited duration of analgesia (12-18 hrs) [64], leading to severe breakthrough pain in the postoperative period and increasing opioid consumption. Thus, the LIA may not be a better alternative to DSB.

Some patients (30 out of 104) in our study developed significant pain postoperatively, requiring rescue analgesics in the form of opioids with or without rescue blocks. We had identified the likely cause of the pain by assessing the type and actual pain location. The most common location of the pain within the six hours of DSB was the proximal thigh, suggesting tourniquet-related pain due to regression of the spinal level. The tourniquet pain, being an inflammatory type, requires perioperative anti-inflammatory pain medications. We found no significant correlations between thigh pain with the duration and pressure of the tourniquet during surgery. The confounding factors affecting the outcome of the block when the likely cause of the pain was an “inadequate block” are the sonoanatomy image quality at the time of the block, difficulty in identifying target structures, and the experience of the regional anesthetist. Our focused pain assessment and determination of the rescue technique as per the pain location (pain location mapping) helped us identify and target specific spared innervations in rescue blocks. The rescue block for Location 2a (upper incision-NVM territory) was FTB targeting only NVM, for Location 2b (lower incision-SN territory) was FTB targeting only SN, and Location 3 (posterior/intraarticular area-popliteal plexus territory) was ACB depositing drug below the VAM. For other locations, only rescue opioids were given without blocks.

The role of opioids in postoperative analgesia is like a double-edged sword: oligoanalgesia at lower doses and analgesia at the expense of potential complications at higher doses. However, lower doses with MMA help reduce undesirable side effects without compromising the analgesic profile. A low maintenance dose of opioids helps manage chronic pain components associated with the osteoarthritic changes in the knee joint prior to the surgery. In our protocol, we included a transdermal patch of buprenorphine 5-10 mg to meet the low maintenance dose of opioids. Our comparative study showed almost no need for additional opioids as rescue analgesia, making DSB an opioid-sparing RA option. It facilitated early discharge from the hospital by avoiding opioid-related side effects while maintaining an effective analgesic profile with MMA. The individual components of DSB (FTB and ACB) are also known to reduce opioid consumption [48,65-67]. Thus, the extended analgesic profile of DSB is the ultimate contribution of the site-specific effect of its components along with the actions of various pharmacological agents used in MMA at all levels of the pain pathway. DSB also helped avoid the use of indwelling catheters by keeping the patient comfortable and pain-free until discharge.

## Conclusions

Perioperative pain management for patients undergoing TKR is complex and challenging. A holistic approach is required to manage such multifactorial postoperative pain taking into account the patient’s age, comorbid factors, complex innervations of pain generators, the demand for the most appropriate analgesic option, enhanced recovery, and early mobilization. Such an approach helped us keep our patients’ overall satisfaction score of 4/5 (very good) at discharge with the existing analgesia protocol.

Despite the strength of our study, including randomization and double-blind design to avoid biases and assessment of all study parameters till the discharge of the patient, our study has several limitations. One of the limitations is its monocentric design and inter-operator variability, potentially leading to inconsistency in the efficacy of the RA performed. Another limitation is the lack of a control group. Although the sample size was calculated statistically, we can not generalize our observations to all TKR surgeries with a limited number of patients included in this study. Unmeasured confounders such as individual muscle mass may have influenced LA spread and motor strength. It would have posed a significant methodological hurdle and external validity problems if we had adjusted anthropometric dimensions to overcome this problem.

In conclusion, DSB with the volumes of 10/20 or 20/10 ml (in FT/AC regions) can provide equivocal and effective postoperative analgesia without compromising the motor strength of the quadriceps muscle, thus allowing implementation of the ERAS protocol for TKR surgery. Correctly applying this novel technique requires focusing on the target structures, using LA at the recommended volume and concentration, and identifying the injection sites as described. Any change to any of this information can lead to unsatisfactory results. This new innovative technique requires prospective multicenter randomized controlled trials with larger samples sizes for better corroboration of the results. Also, the best and optimal volumes for DSB need further study.

## References

[1] Laupattarakasem W, Laopaiboon M, Laupattarakasem P, Sumananont C (2008). Arthroscopic debridement for knee osteoarthritis. Cochrane Database Syst Rev.

[2] (2004). Global Burden of Disease 2004 Update. World Health Organization. Global Burden of Disease Report.

[3] Wittenauer R, Smith L, Aden K (2004). Background Paper 6.12 Osteoarthritis. Background Paper.

[4] (2021). Osteoarthritis. https://medlineplus.gov/ency/article/000423.htm.

[5] Evans JT, Evans JP, Walker RW, Blom AW, Whitehouse MR, Sayers A (2019). How long does a hip replacement last? A systematic review and meta-analysis of case series and national registry reports with more than 15 years of follow-up. Lancet.

[6] Johnson RL, Kopp SL (2014). Optimizing perioperative management of total joint arthroplasty. Anesthesiol Clin.

[7] Chan EY, Fransen M, Parker DA, Assam PN, Chua N (2014). Femoral nerve blocks for acute postoperative pain after knee replacement surgery. Cochrane Database Syst Rev.

[8] Gardner E (1948). The innervation of the hip joint. Anat Rec.

[9] Kennedy JC, Alexander IJ, Hayes KC (1982). Nerve supply of the human knee and its functional importance. Am J Sports Med.

[10] Gray H (1908). Anatomy, Descriptive and Surgical. Philadelphia: Lea & Febiger.

[11] Vas L, Pai R, Khandagale N, Pattnaik M (2014). Pulsed radiofrequency of the composite nerve supply to the knee joint as a new technique for relieving osteoarthritic pain: a preliminary report. Pain Physician.

[12] Horner G, Dellon AL (1994). Innervation of the human knee joint and implications for surgery. Clin Orthop Relat Res.

[13] Tran J, Peng PW, Lam K, Baig E, Agur AM, Gofeld M (2018). Anatomical study of the innervation of anterior knee joint capsule: implication for image-guided intervention. Reg Anesth Pain Med.

[14] Franco CD, Buvanendran A, Petersohn JD, Menzies RD, Menzies LP (2015). Innervation of the anterior capsule of the human knee: implications for radiofrequency ablation. Reg Anesth Pain Med.

[15] Orduña Valls JM, Vallejo R, López Pais P (2017). Anatomic and ultrasonographic evaluation of the knee sensory innervation: a cadaveric study to determine anatomic targets in the treatment of chronic knee pain. Reg Anesth Pain Med.

[16] Fonkoué L, Behets C, Kouassi JK, Coyette M, Detrembleur C, Thienpont E, Cornu O (2019). Distribution of sensory nerves supplying the knee joint capsule and implications for genicular blockade and radiofrequency ablation: an anatomical study. Surg Radiol Anat.

[17] Burckett-St Laurant D, Peng P, Girón Arango L, Niazi AU, Chan VW, Agur A, Perlas A (2016). The nerves of the adductor canal and the innervation of the knee: an anatomic study. Reg Anesth Pain Med.

[18] Kavolus JJ, Sia D, Potter HG, Attarian DE, Lachiewicz PF (2018). Saphenous nerve block from within the knee is feasible for TKA: MRI and cadaveric study. Clin Orthop Relat Res.

[19] Anagnostopoulou S, Anagnostis G, Saranteas T, Mavrogenis AF, Paraskeuopoulos T (2016). Saphenous and infrapatellar nerves at the adductor canal: anatomy and implications in regional anesthesia. Orthopedics.

[20] Hirasawa Y, Okajima S, Ohta M, Tokioka T (2000). Nerve distribution to the human knee joint: anatomical and immunohistochemical study. Int Orthop.

[21] Haus J, Halata Z (1990). Innervation of the anterior cruciate ligament. Int Orthop.

[22] Krauspe R, Schmitz F, Zöller G, Drenckhahn D (1995). Distribution of neurofilament-positive nerve fibres and sensory endings in the human anterior cruciate ligament. Arch Orthop Trauma Surg.

[23] Ikeuchi M, Wang Q, Izumi M, Tani T (2012). Nociceptive sensory innervation of the posterior cruciate ligament in osteoarthritic knees. Arch Orthop Trauma Surg.

[24] Day B, Mackenzie WG, Shim SS, Leung G (1985). The vascular and nerve supply of the human meniscus. Arthroscopy.

[25] Mine T, Kimura M, Sakka A, Kawai S (2000). Innervation of nociceptors in the menisci of the knee joint: an immunohistochemical study. Arch Orthop Trauma Surg.

[26] Sonawane K, Dixit H, Balavenkatasubramanian J, Goel VK (2021). "Dual subsartorial block (DSB)": an innovative procedure-specific, motor-sparing and opioid-sparing regional analgesia technique for total knee replacement surgery - a pilot study. J Clin Anesth.

[27] Sonawane K, Dixit H (2021). Regional analgesia for knee surgeries: thinking beyond borders. Topics in Regional Anesthesia.

[28] Kartik S, Hrudini D, Tuhin M, J B (2021). Anatomical and technical considerations of “dual subsartorial block” (DSB), a novel motor-sparing regional analgesia technique for total knee arthroplasty. Open J Orthop Rheumatol.

[29] Youdas JW, Hollman JH, Hitchcock JR, Hoyme GJ, Johnsen JJ (2007). Comparison of hamstring and quadriceps femoris electromyographic activity between men and women during a single-limb squat on both a stable and labile surface. J Strength Cond Res.

[30] Thacher RR, Hickernell TR, Grosso MJ (2017). Decreased risk of knee buckling with adductor canal block versus femoral nerve block in total knee arthroplasty: a retrospective cohort study. Arthroplast Today.

[31] Sonawane K, Dixit H, Balavenkatasubramanian J (2021). Regional analgesia technique for postoperative analgesia in total knee arthroplasty: have we hit the bull's eye yet?. Braz J Anesthesiol.

[32] Johnston DF, Black ND, Cowden R, Turbitt L, Taylor S (2019). Spread of dye injectate in the distal femoral triangle versus the distal adductor canal: a cadaveric study. Reg Anesth Pain Med.

[33] Kopp SL, Børglum J, Buvanendran A (2017). Anesthesia and analgesia practice pathway options for total knee arthroplasty: an evidence-based review by the American and European Societies of Regional Anesthesia and Pain Medicine. Reg Anesth Pain Med.

[34] Pascarella G, Costa F, Del Buono R, Agrò FE (2019). Adductor canal and femoral triangle: two different rooms with the same door. Saudi J Anaesth.

[35] Swenson JD, Davis JJ, Loose EC (2015). The subsartorial plexus block: a variation on the adductor canal block. Reg Anesth Pain Med.

[36] Andersen HL, Zaric D (2014). Adductor canal block or midthigh saphenous nerve block: same same but different name!. Reg Anesth Pain Med.

[37] Ozer H, Tekdemir I, Elhan A, Turanli S, Engebretsen L (2004). A clinical case and anatomical study of the innervation supply of the vastus medialis muscle. Knee Surg Sports Traumatol Arthrosc.

[38] Andersen HL, Andersen SL, Tranum-Jensen J (2015). The spread of injectate during saphenous nerve block at the adductor canal: a cadaver study. Acta Anaesthesiol Scand.

[39] Runge C, Moriggl B, Børglum J, Bendtsen TF (2017). The spread of ultrasound-guided injectate from the adductor canal to the genicular branch of the posterior obturator nerve and the popliteal plexus: a cadaveric study. Reg Anesth Pain Med.

[40] Bendtsen TF, Moriggl B, Chan V, Børglum J (2016). The optimal analgesic block for total knee arthroplasty. Reg Anesth Pain Med.

[41] Jæger P, Zaric D, Fomsgaard JS (2013). Adductor canal block versus femoral nerve block for analgesia after total knee arthroplasty: a randomized, double-blind study. Reg Anesth Pain Med.

[42] Ghodki PS, Shalu PS, Sardesai SP (2018). Ultrasound-guided adductor canal block versus femoral nerve block for arthroscopic anterior cruciate ligament repair under general anesthesia. J Anaesthesiol Clin Pharmacol.

[43] Kukreja P, Bevinetto C, Brooks B, McKissack H, Montgomery TP, Alexander B, Shah A (2019). Comparison of adductor canal block and femoral nerve block for early ambulation after primary total knee arthroplasty: a randomized controlled trial. Cureus.

[44] Chuan A, Lansdown A, Brick KL (2019). Adductor canal versus femoral triangle anatomical locations for continuous catheter analgesia after total knee arthroplasty: a multicentre randomised controlled study. Br J Anaesth.

[45] Wong WY, Bjørn S, Strid JM, Børglum J, Bendtsen TF (2017). Defining the location of the adductor canal using yltrasound. Reg Anesth Pain Med.

[46] Vora MU, Nicholas TA, Kassel CA, Grant SA (2016). Adductor canal block for knee surgical procedures: review article. J Clin Anesth.

[47] Panchamia JK, Niesen AD, Amundson AW (2018). Adductor canal versus femoral triangle: let us all get on the same page. Anesth Analg.

[48] Wang D, Yang Y, Li Q (2017). Adductor canal block versus femoral nerve block for total knee arthroplasty: a meta-analysis of randomized controlled trials. Sci Rep.

[49] Erskine R (2019). ESRA19-0573 1% 2-chloroprocaine for day case unicompartmental knee replacement. Reg Anesth Pain Med.

[50] Grevstad U, Mathiesen O, Valentiner LS, Jaeger P, Hilsted KL, Dahl JB (2015). Effect of adductor canal block versus femoral nerve block on quadriceps strength, mobilization, and pain after total knee arthroplasty: a randomized, blinded study. Reg Anesth Pain Med.

[51] Johnston DF, Sondekoppam RV, Giffin R, Litchfield R, Ganapathy S (2017). Determination of ED50 and ED95 of 0.5% ropivacaine in adductor canal block to produce quadriceps weakness: a dose-finding study. Reg Anesth Pain Med.

[52] Kuthiala G, Chaudhary G (2011). Ropivacaine: a review of its pharmacology and clinical use. Indian J Anaesth.

[53] Wong AK, Keeney LG, Chen L, Williams R, Liu J, Elkassabany NM (2016). Effect of local anesthetic concentration (0.2% vs 0.1% ropivacaine) On pulmonary function, and analgesia after ultrasound-guided interscalene brachial plexus block: a randomized controlled study. Pain Med.

[54] Choi S, Rodseth R, McCartney CJ (2014). Effects of dexamethasone as a local anaesthetic adjuvant for brachial plexus block: a systematic review and meta-analysis of randomized trials. Br J Anaesth.

[55] Huynh TM, Marret E, Bonnet F (2015). Combination of dexamethasone and local anaesthetic solution in peripheral nerve blocks: a meta-analysis of randomised controlled trials. Eur J Anaesthesiol.

[56] Roy R, Agarwal G, Pradhan C, Kuanar D (2020). Total postoperative analgesia for total knee arthroplasty: ultrasound guided single injection modified 4 in 1 block. J Anaesthesiol Clin Pharmacol.

[57] Thobhani S, Scalercio L, Elliott CE (2017). Novel regional techniques for total knee arthroplasty promote reduced hospital length of stay: an analysis of 106 patients. Ochsner J.

[58] Tran J, Giron Arango L, Peng P, Sinha SK, Agur A, Chan V (2019). Evaluation of the iPACK block injectate spread: a cadaveric study. Reg Anesth Pain Med.

[59] Kerr DR, Kohan L (2008). Local infiltration analgesia: a technique for the control of acute postoperative pain following knee and hip surgery: a case study of 325 patients. Acta Orthop.

[60] Berninger MT, Friederichs J, Leidinger W, Augat P, Bühren V, Fulghum C, Reng W (2018). Effect of local infiltration analgesia, peripheral nerve blocks, general and spinal anesthesia on early functional recovery and pain control in total knee arthroplasty. BMC Musculoskelet Disord.

[61] Desai A, Ramankutty S, Board T, Raut V (2009). Does intraarticular steroid infiltration increase the rate of infection in subsequent total knee replacements?. Knee.

[62] Gulihar A, Robati S, Twaij H, Salih A, Taylor GJ (2015). Articular cartilage and local anaesthetic: a systematic review of the current literature. J Orthop.

[63] Piper SL, Kramer JD, Kim HT, Feeley BT (2011). Effects of local anesthetics on articular cartilage. Am J Sports Med.

[64] Zhang S, Wang F, Lu ZD, Li YP, Zhang L, Jin QH (2011). Effect of single-injection versus continuous local infiltration analgesia after total knee arthroplasty: a randomized, double-blind, placebo-controlled study. J Int Med Res.

[65] Runge C, Børglum J, Jensen JM (2016). The analgesic effect of obturator nerve block added to a femoral triangle block after total knee arthroplasty: a randomized controlled trial. Reg Anesth Pain Med.

[66] Runge C, Jensen JM, Clemmesen L, Knudsen HB, Holm C, Børglum J, Bendtsen TF (2018). Analgesia of combined femoral triangle and obturator nerve blockade is superior to local infiltration analgesia after total knee arthroplasty with high-dose intravenous dexamethasone. Reg Anesth Pain Med.

[67] Hanson NA, Allen CJ, Hostetter LS (2014). Continuous ultrasound-guided adductor canal block for total knee arthroplasty: a randomized, double-blind trial. Anesth Analg.

